# Diaqua­bis­(pyridine-2-carboxyl­ato-κ^2^ *N*,*O*)manganese(II) dimethyl­formamide hemisolvate

**DOI:** 10.1107/S1600536811042218

**Published:** 2011-10-22

**Authors:** Irina A. Golenya, Alexander N. Boyko, Valentina A. Kalibabchuk, Matti Haukka, Stefania V. Tomyn

**Affiliations:** a Kiev National Taras Shevchenko University, Department of Chemistry, Volodymyrska Str. 64, 01601 Kiev, Ukraine; b O.O. Bohomolets National Medical University, Department of General Chemistry, Shevchenko blvd. 13, 01601 Kiev, Ukraine; c University of Joensuu, Department of Chemistry, PO Box 111, FI-80101 Joensuu, Finland

## Abstract

There are two crystallographically independent complex mol­ecules with very similar geometries in the unit cell of the title compound, [Mn(C_6_H_4_NO_2_)_2_(H_2_O)_2_]·0.5C_3_H_7_NO. The central ion is situated in a distorted octa­hedral environment of two N- and four O-donor atoms from two pyridine-2-carboxyl­ate ligands and two *cis*-disposed water mol­ecules. The carboxyl­ate ligands are coordinated in a chelate fashion with the formation of two five-membered rings. In the crystal, the complex mol­ecules are connected by O—H⋯O hydrogen bonds between the coordinated water mol­ecules and the uncoordinated carboxyl­ate O atoms, thus forming hydrogen-bonded walls disposed perpendicularly to the *bc* plane.

## Related literature

For the use of hydroxamate and carboxyl­ate ligands in the synthesis of polynuclear compounds, see: Sliva *et al.* (1997 ▶); Fritsky *et al.* (1998 ▶); Mokhir *et al.* (2002 ▶); Sachse *et al.* (2008 ▶). For hydrolytic destruction of hydroxamate ligands upon complex formation, see: Dobosz *et al.* (1999 ▶); Świątek-Kozłowska *et al.* (2000 ▶). For the synthesis of pyridine-2-hydroxamic acid, see: Hynes (1970 ▶). For related structures, see: Krämer & Fritsky (2000 ▶); Fritsky *et al.* (2001 ▶); Kovbasyuk *et al.* (2004 ▶); Wörl *et al.* (2005*a* ▶,*b* ▶); Moroz *et al.* (2010 ▶).
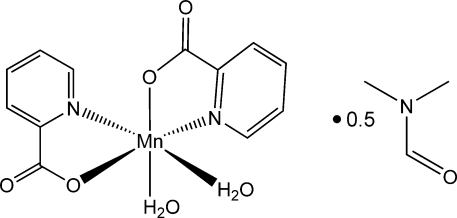


## Experimental

### 

#### Crystal data

[Mn(C_6_H_4_NO_2_)_2_(H_2_O)_2_]·0.5C_3_H_7_NO
*M* *_r_* = 371.73
Triclinic, 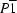

*a* = 8.6860 (17) Å
*b* = 13.532 (3) Å
*c* = 14.871 (3) Å
α = 73.18 (3)°
β = 73.53 (3)°
γ = 72.37 (3)°
*V* = 1557.4 (7) Å^3^
*Z* = 4
Mo *K*α radiation
μ = 0.89 mm^−1^
*T* = 120 K
0.21 × 0.15 × 0.06 mm

#### Data collection

Nonius KappaCCD diffractometer
Absorption correction: multi-scan (*DENZO*/*SCALEPACK*; Otwinowski & Minor, 1997 ▶) *T*_min_ = 0.834, *T*_max_ = 0.932
13428 measured reflections
7243 independent reflections
5370 reflections with *I* > 2σ(*I*)
*R*_int_ = 0.029

#### Refinement

*R*[*F* ^2^ > 2σ(*F* ^2^)] = 0.056
*wR*(*F* ^2^) = 0.157
*S* = 1.04
7243 reflections
426 parameters
H-atom parameters constrained
Δρ_max_ = 1.73 e Å^−3^
Δρ_min_ = −0.87 e Å^−3^

### 

Data collection: *COLLECT* (Nonius, 2000 ▶); cell refinement: *DENZO*/*SCALEPACK* (Otwinowski & Minor, 1997 ▶); data reduction: *DENZO*/*SCALEPACK*; program(s) used to solve structure: *SIR2004* (Burla *et al.*, 2005 ▶); program(s) used to refine structure: *SHELXL97* (Sheldrick, 2008 ▶); molecular graphics: *DIAMOND* (Brandenburg, 2008 ▶); software used to prepare material for publication: *SHELXL97*.

## Supplementary Material

Crystal structure: contains datablock(s) I, global. DOI: 10.1107/S1600536811042218/zl2414sup1.cif

Structure factors: contains datablock(s) I. DOI: 10.1107/S1600536811042218/zl2414Isup2.hkl

Additional supplementary materials:  crystallographic information; 3D view; checkCIF report

## Figures and Tables

**Table 1:** Hydrogen-bond geometry (Å, °)

*D*—H⋯*A*	*D*—H	H⋯*A*	*D*⋯*A*	*D*—H⋯*A*
O1*W*—H1*W*1⋯O7^i^	0.84	1.97	2.729 (3)	150
O1*W*—H1*W*2⋯O6^ii^	0.93	1.76	2.685 (3)	177
O2*W*—H2*W*1⋯O5^ii^	0.84	1.90	2.713 (3)	164
O2*W*—H2*W*2⋯O1^iii^	0.84	1.87	2.700 (3)	168
O3*W*—H3*W*1⋯O1^iii^	0.84	1.92	2.723 (3)	160
O3*W*—H3*W*2⋯O4	0.84	1.85	2.688 (3)	175
O4*W*—H4*W*1⋯O3	0.85	1.89	2.734 (3)	174
O4*W*—H4*W*2⋯O7^iv^	0.85	1.88	2.704 (3)	162

Symmetry codes: (i) 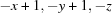; (ii) 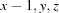; (iii) 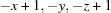; (iv) 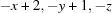.

**Fig. 1.**
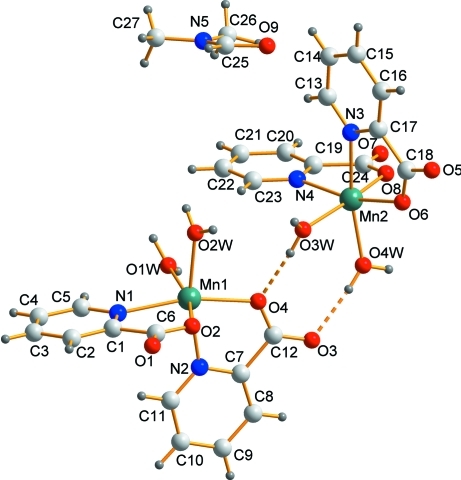
The molecular structure of the title compound showing the atom-numbering scheme employed.

**Fig. 2.**
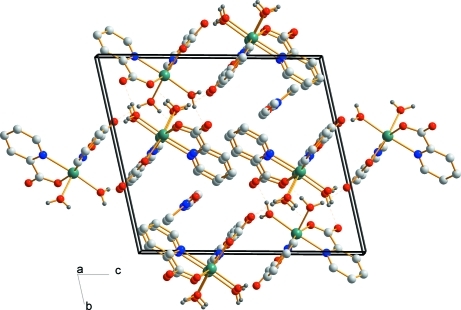
A packing diagram for the title complex. H–atoms not involved in H–bonds are omitted for clarity.

## supplementary crystallographic information

### Comment

Polynuclear complexes based on hydroxamic and carboxylate ligands are widely used in coordination chemistry and molecular magnetism (Sliva *et al.*, 1997; Fritsky *et al.*, 1998; Mokhir *et al.*, 2002; Sachse *et al.*, 2008). In the course of synthesis of polynuclear compounds, the hydroxamic functions (especially those neighboring with adjacent alternative donor groups) sometimes undergo hydrolytic destruction (Dobosz *et al.*, 1999; Świątek-Kozłowska *et al.*, 2000). The title compound was obtained as a result of hydrolytic decomposition of pyridine-2-hydroxamic acid by reaction with manganese(III) acetate.

The central ion of the title compound is situated in a distorted octahedral environment of two N and four O donor atoms from two pyridine-2-carboxylates and two *cis*-disposed water molecules (Fig. 1). The carboxylate ligands are coordinated in a chelate fashion with formation of two five-membered rings.

The C—O bond lengths in the carboxylic moieties differ insignificantly which is normal for monodentately coordinated carboxylates (Wörl *et al.*, 2005*a*,*b*). The C—C and C—N bond lengths in the pyridine rings exhibit normal values (Krämer & Fritsky, 2000; Fritsky *et al.*, 2001; Kovbasyuk *et al.*, 2004; Moroz *et al.*, 2010).

In the crystal neighboring complex molecules are connected through H-bonds between the coordinated water molecules and the non-coordinated carboxylic O atoms thus forming H-bonded walls disposed perpendicularly to the yz plane (Fig.2).

### Experimental

Manganese(III) acetate dihydrate (0.0268 g, 0.1 mmol) was dissolved in water (3 ml) and mixed with a solution of pyridine-2-hydroxamic acid (0.0414 g, 0.3 mmol) (Hynes, 1970) in methanol (3 ml). The mixture was stirred for 30 min. and filtered. The insoluble residue was dissolved in DMF (3 ml) and set aside for crystallization by slow diffusion of methyl tert-buthyl ether vapours to the formed solution. The light-yellow crystals that formed in 5-7 days were filtered off, washed with methyl tert-buthyl ether and dried. Yield 74%. Elemental analysis calc.(%) for C_27_H_31_Mn_2_N_5_O_13_: C 43.62; H 4.20; N 9.42; Mn 14.78; found: C 43.86; H 4.12; N 9.29; Mn 15.01.

### Refinement

Water O—H hydrogen atoms were located from a difference Fourier map. In the final refinement cycles they were constrained to ride on the parent atoms with *U*_iso_ = 1.5 *U*_eq_(parent atom). The remaining H atoms were positioned geometrically and were constrained to ride on their parent atoms with C—H = 0.95–0.987 Å, and with *U*_iso_ = 1.2–1.5 *U*_eq_(parent atom).

### Crystal data

**Table:**

[Mn(C_6_H_4_NO_2_)_2_(H_2_O)_2_]·0.5C_3_H_7_NO	*Z* = 4
*M**_r_* = 371.73	*F*(000) = 764
Triclinic, *P*1	*D*_x_ = 1.585 Mg m^−^^3^
Hall symbol: -P 1	Mo *K*α radiation, λ = 0.71073 Å
*a* = 8.6860 (17) Å	Cell parameters from 4574 reflections
*b* = 13.532 (3) Å	θ = 3.0–27.5°
*c* = 14.871 (3) Å	µ = 0.89 mm^−^^1^
α = 73.18 (3)°	*T* = 120 K
β = 73.53 (3)°	Block, pale yellow
γ = 72.37 (3)°	0.21 × 0.15 × 0.06 mm
*V* = 1557.4 (7) Å^3^	

### Data collection

**Table:**

Nonius KappaCCD diffractometer	7243 independent reflections
Radiation source: fine-focus sealed tube	5370 reflections with *I* > 2σ(*I*)
horizontally mounted graphite crystal	*R*_int_ = 0.029
Detector resolution: 9 pixels mm^-1^	θ_max_ = 28.8°, θ_min_ = 2.9°
φ scans and ω scans with κ offset	*h* = −11→11
Absorption correction: multi-scan (*DENZO*/*SCALEPACK*; Otwinowski & Minor, 1997)	*k* = −17→18
*T*_min_ = 0.834, *T*_max_ = 0.932	*l* = −19→19
13428 measured reflections	

### Refinement

**Table:**

Refinement on *F*^2^	Primary atom site location: structure-invariant direct methods
Least-squares matrix: full	Secondary atom site location: difference Fourier map
*R*[*F*^2^ > 2σ(*F*^2^)] = 0.056	Hydrogen site location: inferred from neighbouring sites
*wR*(*F*^2^) = 0.157	H-atom parameters constrained
*S* = 1.04	*w* = 1/[σ^2^(*F*_o_^2^) + (0.1017*P*)^2^] where *P* = (*F*_o_^2^ + 2*F*_c_^2^)/3
7243 reflections	(Δ/σ)_max_ < 0.001
426 parameters	Δρ_max_ = 1.73 e Å^−^^3^
0 restraints	Δρ_min_ = −0.87 e Å^−^^3^

### Special details

**Table:**

Geometry. All esds (except the esd in the dihedral angle between two l.s. planes) are estimated using the full covariance matrix. The cell esds are taken into account individually in the estimation of esds in distances, angles and torsion angles; correlations between esds in cell parameters are only used when they are defined by crystal symmetry. An approximate (isotropic) treatment of cell esds is used for estimating esds involving l.s. planes.
Refinement. Refinement of F^2^ against ALL reflections. The weighted R-factor wR and goodness of fit S are based on F^2^, conventional R-factors R are based on F, with F set to zero for negative F^2^. The threshold expression of F^2^ > 2sigma(F^2^) is used only for calculating R-factors(gt) etc. and is not relevant to the choice of reflections for refinement. R-factors based on F^2^ are statistically about twice as large as those based on F, and R- factors based on ALL data will be even larger.

### Fractional atomic coordinates and isotropic or equivalent isotropic displacement parameters (Å^2^)

**Table:**

	*x*	*y*	*z*	*U*_iso_*/*U*_eq_	
Mn1	0.41145 (6)	0.09669 (4)	0.28566 (3)	0.01596 (13)	
O1	0.4153 (3)	−0.16990 (17)	0.51623 (15)	0.0198 (5)	
O2	0.4925 (3)	−0.03998 (17)	0.39416 (15)	0.0197 (5)	
O3	0.8614 (3)	0.13230 (19)	0.08858 (18)	0.0284 (5)	
O4	0.6391 (3)	0.14520 (17)	0.21013 (15)	0.0202 (5)	
O1W	0.2631 (3)	0.22912 (17)	0.20112 (15)	0.0234 (5)	
H1W1	0.2914	0.2536	0.1417	0.035*	
H1W2	0.1736	0.2753	0.2312	0.035*	
O2W	0.3491 (3)	0.19490 (18)	0.38793 (16)	0.0225 (5)	
H2W1	0.2781	0.2528	0.3900	0.034*	
H2W2	0.4144	0.1814	0.4245	0.034*	
N1	0.1996 (3)	0.0162 (2)	0.34259 (17)	0.0178 (5)	
N2	0.5325 (3)	0.0018 (2)	0.16917 (18)	0.0182 (5)	
C1	0.2246 (4)	−0.0686 (2)	0.4167 (2)	0.0168 (6)	
C2	0.1082 (4)	−0.1278 (3)	0.4631 (2)	0.0230 (7)	
H2	0.1292	−0.1872	0.5147	0.028*	
C3	−0.0404 (4)	−0.0984 (3)	0.4327 (2)	0.0258 (7)	
H3	−0.1225	−0.1376	0.4636	0.031*	
C4	−0.0671 (4)	−0.0119 (3)	0.3574 (2)	0.0238 (7)	
H4	−0.1675	0.0094	0.3356	0.029*	
C5	0.0555 (4)	0.0434 (3)	0.3142 (2)	0.0217 (7)	
H5	0.0370	0.1030	0.2623	0.026*	
C6	0.3907 (4)	−0.0943 (2)	0.4446 (2)	0.0180 (6)	
C7	0.6736 (4)	0.0260 (2)	0.1121 (2)	0.0181 (6)	
C8	0.7645 (4)	−0.0236 (3)	0.0377 (2)	0.0221 (7)	
H8	0.8622	−0.0039	−0.0021	0.027*	
C9	0.7103 (4)	−0.1031 (3)	0.0222 (2)	0.0231 (7)	
H9	0.7702	−0.1383	−0.0286	0.028*	
C10	0.5679 (4)	−0.1299 (3)	0.0819 (2)	0.0245 (7)	
H10	0.5294	−0.1847	0.0736	0.029*	
C11	0.4819 (4)	−0.0751 (2)	0.1546 (2)	0.0211 (6)	
H11	0.3836	−0.0931	0.1953	0.025*	
C12	0.7307 (4)	0.1088 (2)	0.1381 (2)	0.0183 (6)	
Mn2	0.79814 (5)	0.40535 (4)	0.21576 (3)	0.01583 (13)	
O5	1.0811 (3)	0.35722 (19)	0.42074 (17)	0.0279 (5)	
O6	0.9995 (3)	0.35619 (17)	0.29141 (15)	0.0196 (5)	
O7	0.7687 (3)	0.66837 (17)	−0.01771 (15)	0.0196 (5)	
O8	0.8505 (3)	0.54221 (17)	0.10592 (15)	0.0188 (4)	
O3W	0.6997 (3)	0.27064 (17)	0.30053 (15)	0.0234 (5)	
H3W1	0.6725	0.2504	0.3606	0.035*	
H3W2	0.6863	0.2297	0.2718	0.035*	
O4W	0.9233 (3)	0.30931 (18)	0.11082 (16)	0.0226 (5)	
H4W1	0.9111	0.2518	0.1054	0.034*	
H4W2	1.0101	0.3263	0.0727	0.034*	
N3	0.7155 (3)	0.4956 (2)	0.33415 (18)	0.0174 (5)	
N4	0.5622 (3)	0.4837 (2)	0.16008 (17)	0.0177 (5)	
C13	0.5772 (4)	0.5718 (2)	0.3498 (2)	0.0208 (6)	
H13	0.5033	0.5917	0.3077	0.025*	
C14	0.5367 (4)	0.6234 (3)	0.4250 (2)	0.0225 (7)	
H14	0.4383	0.6781	0.4332	0.027*	
C15	0.6425 (4)	0.5936 (3)	0.4874 (2)	0.0236 (7)	
H15	0.6168	0.6264	0.5401	0.028*	
C16	0.7875 (4)	0.5145 (3)	0.4719 (2)	0.0216 (6)	
H16	0.8622	0.4921	0.5138	0.026*	
C17	0.8198 (4)	0.4693 (2)	0.3938 (2)	0.0181 (6)	
C18	0.9806 (4)	0.3871 (2)	0.3680 (2)	0.0197 (6)	
C19	0.5778 (4)	0.5680 (2)	0.0848 (2)	0.0165 (6)	
C20	0.4495 (4)	0.6252 (2)	0.0383 (2)	0.0204 (6)	
H20	0.4640	0.6836	−0.0146	0.024*	
C21	0.2996 (4)	0.5961 (3)	0.0700 (2)	0.0238 (7)	
H21	0.2100	0.6341	0.0392	0.029*	
C22	0.2830 (4)	0.5102 (3)	0.1475 (2)	0.0236 (7)	
H22	0.1815	0.4889	0.1709	0.028*	
C23	0.4172 (4)	0.4558 (3)	0.1901 (2)	0.0213 (6)	
H23	0.4058	0.3966	0.2425	0.026*	
C24	0.7461 (4)	0.5947 (2)	0.0553 (2)	0.0175 (6)	
O9	0.2692 (4)	0.7270 (3)	0.2913 (3)	0.0718 (12)	
N5	0.0270 (4)	0.7605 (3)	0.2458 (2)	0.0367 (8)	
C25	0.1253 (6)	0.7140 (4)	0.3062 (4)	0.0586 (14)	
H25	0.0863	0.6681	0.3644	0.070*	
C26	0.0813 (5)	0.8336 (4)	0.1558 (3)	0.0434 (10)	
H26A	0.0243	0.9070	0.1607	0.065*	
H26B	0.0550	0.8178	0.1024	0.065*	
H26C	0.2010	0.8249	0.1442	0.065*	
C27	−0.1407 (6)	0.7521 (5)	0.2627 (4)	0.078 (2)	
H27A	−0.1711	0.7089	0.3274	0.117*	
H27B	−0.1499	0.7184	0.2150	0.117*	
H27C	−0.2152	0.8233	0.2574	0.117*	

### Atomic displacement parameters (Å^2^)

**Table:**

	*U*^11^	*U*^22^	*U*^33^	*U*^12^	*U*^13^	*U*^23^
Mn1	0.0194 (2)	0.0161 (2)	0.0105 (2)	−0.00402 (18)	−0.00322 (17)	−0.00040 (17)
O1	0.0257 (11)	0.0179 (11)	0.0129 (10)	−0.0041 (9)	−0.0060 (9)	0.0016 (8)
O2	0.0207 (11)	0.0202 (11)	0.0167 (11)	−0.0061 (9)	−0.0060 (9)	0.0013 (9)
O3	0.0245 (12)	0.0319 (13)	0.0314 (13)	−0.0125 (10)	0.0067 (10)	−0.0172 (11)
O4	0.0245 (11)	0.0213 (11)	0.0153 (11)	−0.0066 (9)	−0.0014 (9)	−0.0066 (9)
O1W	0.0281 (12)	0.0219 (12)	0.0109 (10)	0.0021 (9)	−0.0033 (9)	0.0008 (8)
O2W	0.0215 (11)	0.0256 (12)	0.0211 (12)	0.0012 (9)	−0.0077 (9)	−0.0103 (9)
N1	0.0212 (13)	0.0184 (13)	0.0119 (12)	−0.0023 (10)	−0.0043 (10)	−0.0022 (10)
N2	0.0191 (13)	0.0192 (13)	0.0156 (13)	−0.0035 (10)	−0.0044 (10)	−0.0036 (10)
C1	0.0202 (14)	0.0184 (15)	0.0112 (14)	−0.0042 (12)	−0.0015 (11)	−0.0046 (11)
C2	0.0285 (17)	0.0224 (16)	0.0152 (15)	−0.0073 (13)	−0.0027 (13)	−0.0002 (12)
C3	0.0254 (17)	0.0305 (19)	0.0225 (17)	−0.0121 (14)	−0.0017 (13)	−0.0051 (14)
C4	0.0230 (16)	0.0295 (18)	0.0189 (16)	−0.0066 (14)	−0.0048 (13)	−0.0051 (13)
C5	0.0255 (16)	0.0253 (17)	0.0121 (14)	−0.0066 (13)	−0.0046 (12)	0.0000 (12)
C6	0.0258 (16)	0.0178 (15)	0.0101 (14)	−0.0030 (12)	−0.0037 (12)	−0.0050 (11)
C7	0.0213 (15)	0.0174 (15)	0.0158 (15)	−0.0034 (12)	−0.0053 (12)	−0.0040 (11)
C8	0.0216 (15)	0.0250 (17)	0.0182 (16)	−0.0008 (13)	−0.0041 (12)	−0.0079 (13)
C9	0.0249 (16)	0.0215 (16)	0.0240 (17)	−0.0004 (13)	−0.0065 (13)	−0.0108 (13)
C10	0.0295 (17)	0.0219 (17)	0.0262 (18)	−0.0063 (13)	−0.0094 (14)	−0.0083 (13)
C11	0.0220 (15)	0.0194 (15)	0.0222 (16)	−0.0060 (12)	−0.0047 (12)	−0.0039 (12)
C12	0.0204 (15)	0.0164 (15)	0.0174 (15)	−0.0031 (12)	−0.0034 (12)	−0.0048 (12)
Mn2	0.0197 (2)	0.0157 (2)	0.0107 (2)	−0.00441 (18)	−0.00352 (17)	−0.00042 (17)
O5	0.0278 (12)	0.0315 (13)	0.0285 (13)	0.0045 (10)	−0.0161 (10)	−0.0150 (11)
O6	0.0217 (11)	0.0216 (11)	0.0150 (11)	−0.0018 (9)	−0.0039 (8)	−0.0071 (9)
O7	0.0253 (11)	0.0184 (11)	0.0127 (10)	−0.0069 (9)	−0.0027 (8)	0.0004 (8)
O8	0.0203 (11)	0.0195 (11)	0.0144 (11)	−0.0071 (9)	−0.0041 (8)	0.0024 (8)
O3W	0.0395 (13)	0.0236 (12)	0.0099 (10)	−0.0171 (10)	−0.0037 (9)	0.0005 (8)
O4W	0.0243 (12)	0.0240 (12)	0.0209 (12)	−0.0108 (9)	0.0016 (9)	−0.0080 (9)
N3	0.0197 (12)	0.0154 (12)	0.0166 (13)	−0.0046 (10)	−0.0045 (10)	−0.0019 (10)
N4	0.0228 (13)	0.0174 (13)	0.0122 (12)	−0.0050 (10)	−0.0047 (10)	−0.0012 (10)
C13	0.0219 (15)	0.0202 (16)	0.0201 (16)	−0.0037 (12)	−0.0069 (12)	−0.0033 (12)
C14	0.0218 (16)	0.0173 (15)	0.0261 (17)	−0.0018 (12)	−0.0030 (13)	−0.0066 (13)
C15	0.0261 (16)	0.0248 (17)	0.0208 (16)	−0.0088 (13)	0.0009 (13)	−0.0093 (13)
C16	0.0231 (16)	0.0234 (16)	0.0204 (16)	−0.0052 (13)	−0.0057 (13)	−0.0073 (13)
C17	0.0203 (15)	0.0190 (15)	0.0144 (14)	−0.0051 (12)	−0.0041 (12)	−0.0022 (12)
C18	0.0218 (15)	0.0161 (15)	0.0215 (16)	−0.0051 (12)	−0.0044 (12)	−0.0044 (12)
C19	0.0249 (15)	0.0152 (14)	0.0076 (13)	−0.0036 (12)	−0.0034 (11)	−0.0012 (11)
C20	0.0276 (16)	0.0183 (15)	0.0151 (15)	−0.0049 (13)	−0.0066 (12)	−0.0021 (12)
C21	0.0219 (16)	0.0253 (17)	0.0252 (17)	−0.0011 (13)	−0.0096 (13)	−0.0074 (13)
C22	0.0200 (15)	0.0295 (18)	0.0225 (17)	−0.0088 (13)	−0.0014 (13)	−0.0079 (14)
C23	0.0242 (16)	0.0212 (16)	0.0176 (15)	−0.0053 (13)	−0.0043 (12)	−0.0031 (12)
C24	0.0237 (15)	0.0167 (15)	0.0110 (14)	−0.0041 (12)	−0.0019 (11)	−0.0039 (11)
O9	0.048 (2)	0.081 (3)	0.097 (3)	0.0190 (18)	−0.046 (2)	−0.042 (2)
N5	0.0288 (16)	0.046 (2)	0.0344 (18)	−0.0097 (14)	−0.0108 (14)	−0.0021 (15)
C25	0.050 (3)	0.063 (3)	0.056 (3)	0.018 (2)	−0.027 (2)	−0.021 (3)
C26	0.036 (2)	0.048 (3)	0.043 (2)	−0.0126 (19)	−0.0038 (18)	−0.006 (2)
C27	0.046 (3)	0.075 (4)	0.098 (5)	−0.037 (3)	−0.027 (3)	0.041 (3)

### Geometric parameters (Å, °)

**Table:**

Mn1—O2W	2.154 (2)	O5—C18	1.237 (4)
Mn1—O2	2.156 (2)	O6—C18	1.276 (4)
Mn1—O1W	2.161 (2)	O7—C24	1.257 (4)
Mn1—O4	2.168 (2)	O8—C24	1.257 (4)
Mn1—N1	2.256 (3)	O3W—H3W1	0.8410
Mn1—N2	2.279 (3)	O3W—H3W2	0.8406
O1—C6	1.262 (4)	O4W—H4W1	0.8452
O2—C6	1.259 (4)	O4W—H4W2	0.8548
O3—C12	1.241 (4)	N3—C13	1.338 (4)
O4—C12	1.268 (4)	N3—C17	1.346 (4)
O1W—H1W1	0.8405	N4—C23	1.341 (4)
O1W—H1W2	0.9305	N4—C19	1.357 (4)
O2W—H2W1	0.8402	C13—C14	1.394 (4)
O2W—H2W2	0.8402	C13—H13	0.9500
N1—C5	1.346 (4)	C14—C15	1.382 (5)
N1—C1	1.353 (4)	C14—H14	0.9500
N2—C11	1.335 (4)	C15—C16	1.395 (4)
N2—C7	1.350 (4)	C15—H15	0.9500
C1—C2	1.384 (4)	C16—C17	1.388 (4)
C1—C6	1.525 (4)	C16—H16	0.9500
C2—C3	1.395 (5)	C17—C18	1.529 (4)
C2—H2	0.9500	C19—C20	1.387 (4)
C3—C4	1.380 (5)	C19—C24	1.525 (4)
C3—H3	0.9500	C20—C21	1.389 (5)
C4—C5	1.387 (5)	C20—H20	0.9500
C4—H4	0.9500	C21—C22	1.390 (5)
C5—H5	0.9500	C21—H21	0.9500
C7—C8	1.382 (4)	C22—C23	1.390 (4)
C7—C12	1.532 (4)	C22—H22	0.9500
C8—C9	1.395 (4)	C23—H23	0.9500
C8—H8	0.9500	O9—C25	1.263 (6)
C9—C10	1.383 (5)	N5—C25	1.309 (5)
C9—H9	0.9500	N5—C27	1.440 (5)
C10—C11	1.396 (4)	N5—C26	1.466 (5)
C10—H10	0.9500	C25—H25	0.9500
C11—H11	0.9500	C26—H26A	0.9800
Mn2—O8	2.155 (2)	C26—H26B	0.9800
Mn2—O3W	2.156 (2)	C26—H26C	0.9800
Mn2—O4W	2.159 (2)	C27—H27A	0.9800
Mn2—O6	2.179 (2)	C27—H27B	0.9800
Mn2—N3	2.259 (3)	C27—H27C	0.9800
Mn2—N4	2.269 (3)		
			
O2W—Mn1—O2	89.21 (9)	O6—Mn2—N3	73.39 (9)
O2W—Mn1—O1W	86.34 (9)	O8—Mn2—N4	74.84 (9)
O2—Mn1—O1W	163.98 (9)	O3W—Mn2—N4	90.88 (9)
O2W—Mn1—O4	94.13 (9)	O4W—Mn2—N4	97.90 (9)
O2—Mn1—O4	102.52 (9)	O6—Mn2—N4	166.54 (9)
O1W—Mn1—O4	93.15 (9)	N3—Mn2—N4	93.21 (9)
O2W—Mn1—N1	99.52 (9)	C18—O6—Mn2	120.26 (19)
O2—Mn1—N1	75.12 (9)	C24—O8—Mn2	118.9 (2)
O1W—Mn1—N1	90.44 (9)	Mn2—O3W—H3W1	129.6
O4—Mn1—N1	166.09 (9)	Mn2—O3W—H3W2	118.4
O2W—Mn1—N2	167.35 (9)	H3W1—O3W—H3W2	112.0
O2—Mn1—N2	90.93 (9)	Mn2—O4W—H4W1	131.6
O1W—Mn1—N2	96.76 (9)	Mn2—O4W—H4W2	115.2
O4—Mn1—N2	73.50 (9)	H4W1—O4W—H4W2	112.5
N1—Mn1—N2	92.74 (9)	C13—N3—C17	117.8 (3)
C6—O2—Mn1	118.29 (19)	C13—N3—Mn2	127.1 (2)
C12—O4—Mn1	121.08 (19)	C17—N3—Mn2	115.1 (2)
Mn1—O1W—H1W1	125.2	C23—N4—C19	118.4 (3)
Mn1—O1W—H1W2	120.2	C23—N4—Mn2	128.8 (2)
H1W1—O1W—H1W2	112.7	C19—N4—Mn2	112.9 (2)
Mn1—O2W—H2W1	128.7	N3—C13—C14	123.0 (3)
Mn1—O2W—H2W2	117.1	N3—C13—H13	118.5
H2W1—O2W—H2W2	113.0	C14—C13—H13	118.5
C5—N1—C1	118.2 (3)	C15—C14—C13	118.7 (3)
C5—N1—Mn1	128.6 (2)	C15—C14—H14	120.6
C1—N1—Mn1	113.12 (19)	C13—C14—H14	120.6
C11—N2—C7	118.5 (3)	C14—C15—C16	119.0 (3)
C11—N2—Mn1	127.2 (2)	C14—C15—H15	120.5
C7—N2—Mn1	114.2 (2)	C16—C15—H15	120.5
N1—C1—C2	122.3 (3)	C17—C16—C15	118.4 (3)
N1—C1—C6	115.1 (3)	C17—C16—H16	120.8
C2—C1—C6	122.6 (3)	C15—C16—H16	120.8
C1—C2—C3	118.7 (3)	N3—C17—C16	123.1 (3)
C1—C2—H2	120.6	N3—C17—C18	115.2 (3)
C3—C2—H2	120.6	C16—C17—C18	121.7 (3)
C4—C3—C2	119.3 (3)	O5—C18—O6	125.6 (3)
C4—C3—H3	120.3	O5—C18—C17	118.8 (3)
C2—C3—H3	120.3	O6—C18—C17	115.6 (3)
C3—C4—C5	118.7 (3)	N4—C19—C20	122.1 (3)
C3—C4—H4	120.7	N4—C19—C24	115.1 (3)
C5—C4—H4	120.7	C20—C19—C24	122.7 (3)
N1—C5—C4	122.7 (3)	C19—C20—C21	119.2 (3)
N1—C5—H5	118.6	C19—C20—H20	120.4
C4—C5—H5	118.6	C21—C20—H20	120.4
O2—C6—O1	125.3 (3)	C20—C21—C22	118.8 (3)
O2—C6—C1	117.9 (3)	C20—C21—H21	120.6
O1—C6—C1	116.8 (3)	C22—C21—H21	120.6
N2—C7—C8	122.5 (3)	C21—C22—C23	118.9 (3)
N2—C7—C12	115.5 (3)	C21—C22—H22	120.5
C8—C7—C12	122.0 (3)	C23—C22—H22	120.5
C7—C8—C9	118.8 (3)	N4—C23—C22	122.6 (3)
C7—C8—H8	120.6	N4—C23—H23	118.7
C9—C8—H8	120.6	C22—C23—H23	118.7
C10—C9—C8	118.9 (3)	O7—C24—O8	125.3 (3)
C10—C9—H9	120.6	O7—C24—C19	117.0 (3)
C8—C9—H9	120.6	O8—C24—C19	117.7 (3)
C9—C10—C11	118.8 (3)	C25—N5—C27	125.1 (4)
C9—C10—H10	120.6	C25—N5—C26	119.9 (4)
C11—C10—H10	120.6	C27—N5—C26	114.8 (3)
N2—C11—C10	122.5 (3)	O9—C25—N5	123.6 (5)
N2—C11—H11	118.8	O9—C25—H25	118.2
C10—C11—H11	118.8	N5—C25—H25	118.2
O3—C12—O4	126.6 (3)	N5—C26—H26A	109.5
O3—C12—C7	117.8 (3)	N5—C26—H26B	109.5
O4—C12—C7	115.6 (3)	H26A—C26—H26B	109.5
O8—Mn2—O3W	163.73 (8)	N5—C26—H26C	109.5
O8—Mn2—O4W	88.27 (9)	H26A—C26—H26C	109.5
O3W—Mn2—O4W	86.00 (9)	H26B—C26—H26C	109.5
O8—Mn2—O6	103.96 (9)	N5—C27—H27A	109.5
O3W—Mn2—O6	91.74 (9)	N5—C27—H27B	109.5
O4W—Mn2—O6	95.46 (9)	H27A—C27—H27B	109.5
O8—Mn2—N3	92.93 (9)	N5—C27—H27C	109.5
O3W—Mn2—N3	95.66 (9)	H27A—C27—H27C	109.5
O4W—Mn2—N3	168.75 (9)	H27B—C27—H27C	109.5
			
O2W—Mn1—O2—C6	93.4 (2)	O3W—Mn2—O6—C18	−88.6 (2)
O1W—Mn1—O2—C6	19.6 (4)	O4W—Mn2—O6—C18	−174.8 (2)
O4—Mn1—O2—C6	−172.5 (2)	N3—Mn2—O6—C18	6.7 (2)
N1—Mn1—O2—C6	−6.7 (2)	N4—Mn2—O6—C18	12.5 (5)
N2—Mn1—O2—C6	−99.2 (2)	O3W—Mn2—O8—C24	21.9 (4)
O2W—Mn1—O4—C12	−179.7 (2)	O4W—Mn2—O8—C24	91.2 (2)
O2—Mn1—O4—C12	90.2 (2)	O6—Mn2—O8—C24	−173.6 (2)
O1W—Mn1—O4—C12	−93.1 (2)	N3—Mn2—O8—C24	−100.0 (2)
N1—Mn1—O4—C12	11.6 (5)	N4—Mn2—O8—C24	−7.4 (2)
N2—Mn1—O4—C12	3.0 (2)	O8—Mn2—N3—C13	70.6 (3)
O2W—Mn1—N1—C5	95.5 (3)	O3W—Mn2—N3—C13	−95.6 (3)
O2—Mn1—N1—C5	−177.9 (3)	O4W—Mn2—N3—C13	166.4 (4)
O1W—Mn1—N1—C5	9.2 (3)	O6—Mn2—N3—C13	174.3 (3)
O4—Mn1—N1—C5	−95.9 (4)	N4—Mn2—N3—C13	−4.4 (3)
N2—Mn1—N1—C5	−87.6 (3)	O8—Mn2—N3—C17	−108.6 (2)
O2W—Mn1—N1—C1	−82.5 (2)	O3W—Mn2—N3—C17	85.2 (2)
O2—Mn1—N1—C1	4.08 (19)	O4W—Mn2—N3—C17	−12.8 (6)
O1W—Mn1—N1—C1	−168.9 (2)	O6—Mn2—N3—C17	−4.9 (2)
O4—Mn1—N1—C1	86.1 (4)	N4—Mn2—N3—C17	176.4 (2)
N2—Mn1—N1—C1	94.3 (2)	O8—Mn2—N4—C23	−177.4 (3)
O2W—Mn1—N2—C11	162.7 (4)	O3W—Mn2—N4—C23	10.5 (3)
O2—Mn1—N2—C11	72.1 (3)	O4W—Mn2—N4—C23	96.6 (3)
O1W—Mn1—N2—C11	−93.8 (3)	O6—Mn2—N4—C23	−90.7 (5)
O4—Mn1—N2—C11	174.9 (3)	N3—Mn2—N4—C23	−85.2 (3)
N1—Mn1—N2—C11	−3.0 (3)	O8—Mn2—N4—C19	4.44 (19)
O2W—Mn1—N2—C7	−15.0 (5)	O3W—Mn2—N4—C19	−167.7 (2)
O2—Mn1—N2—C7	−105.6 (2)	O4W—Mn2—N4—C19	−81.6 (2)
O1W—Mn1—N2—C7	88.5 (2)	O6—Mn2—N4—C19	91.1 (4)
O4—Mn1—N2—C7	−2.8 (2)	N3—Mn2—N4—C19	96.6 (2)
N1—Mn1—N2—C7	179.3 (2)	C17—N3—C13—C14	−0.7 (5)
C5—N1—C1—C2	0.5 (4)	Mn2—N3—C13—C14	−179.9 (2)
Mn1—N1—C1—C2	178.7 (2)	N3—C13—C14—C15	−1.1 (5)
C5—N1—C1—C6	−180.0 (3)	C13—C14—C15—C16	1.3 (5)
Mn1—N1—C1—C6	−1.7 (3)	C14—C15—C16—C17	0.3 (5)
N1—C1—C2—C3	−0.5 (5)	C13—N3—C17—C16	2.4 (5)
C6—C1—C2—C3	−180.0 (3)	Mn2—N3—C17—C16	−178.3 (2)
C1—C2—C3—C4	0.2 (5)	C13—N3—C17—C18	−176.1 (3)
C2—C3—C4—C5	0.0 (5)	Mn2—N3—C17—C18	3.1 (3)
C1—N1—C5—C4	−0.2 (5)	C15—C16—C17—N3	−2.2 (5)
Mn1—N1—C5—C4	−178.2 (2)	C15—C16—C17—C18	176.2 (3)
C3—C4—C5—N1	0.0 (5)	Mn2—O6—C18—O5	173.7 (3)
Mn1—O2—C6—O1	−173.0 (2)	Mn2—O6—C18—C17	−7.3 (3)
Mn1—O2—C6—C1	8.0 (3)	N3—C17—C18—O5	−178.5 (3)
N1—C1—C6—O2	−4.0 (4)	C16—C17—C18—O5	3.0 (5)
C2—C1—C6—O2	175.5 (3)	N3—C17—C18—O6	2.4 (4)
N1—C1—C6—O1	176.9 (2)	C16—C17—C18—O6	−176.1 (3)
C2—C1—C6—O1	−3.5 (4)	C23—N4—C19—C20	−0.3 (4)
C11—N2—C7—C8	1.9 (4)	Mn2—N4—C19—C20	178.1 (2)
Mn1—N2—C7—C8	179.8 (2)	C23—N4—C19—C24	179.9 (2)
C11—N2—C7—C12	−175.4 (3)	Mn2—N4—C19—C24	−1.8 (3)
Mn1—N2—C7—C12	2.4 (3)	N4—C19—C20—C21	0.5 (4)
N2—C7—C8—C9	−1.3 (5)	C24—C19—C20—C21	−179.7 (3)
C12—C7—C8—C9	175.9 (3)	C19—C20—C21—C22	0.0 (5)
C7—C8—C9—C10	−0.3 (5)	C20—C21—C22—C23	−0.5 (5)
C8—C9—C10—C11	1.1 (5)	C19—N4—C23—C22	−0.3 (4)
C7—N2—C11—C10	−1.0 (5)	Mn2—N4—C23—C22	−178.4 (2)
Mn1—N2—C11—C10	−178.6 (2)	C21—C22—C23—N4	0.7 (5)
C9—C10—C11—N2	−0.5 (5)	Mn2—O8—C24—O7	−171.5 (2)
Mn1—O4—C12—O3	178.6 (3)	Mn2—O8—C24—C19	9.0 (3)
Mn1—O4—C12—C7	−2.7 (3)	N4—C19—C24—O7	175.9 (3)
N2—C7—C12—O3	178.8 (3)	C20—C19—C24—O7	−3.9 (4)
C8—C7—C12—O3	1.5 (4)	N4—C19—C24—O8	−4.5 (4)
N2—C7—C12—O4	0.0 (4)	C20—C19—C24—O8	175.6 (3)
C8—C7—C12—O4	−177.4 (3)	C27—N5—C25—O9	−176.4 (5)
O8—Mn2—O6—C18	95.6 (2)	C26—N5—C25—O9	−1.2 (7)

### Hydrogen-bond geometry (Å, °)

**Table:**

*D*—H···*A*	*D*—H	H···*A*	*D*···*A*	*D*—H···*A*
O1W—H1W1···O7^i^	0.84	1.97	2.729 (3)	150.
O1W—H1W2···O6^ii^	0.93	1.76	2.685 (3)	177.
O2W—H2W1···O5^ii^	0.84	1.90	2.713 (3)	164.
O2W—H2W2···O1^iii^	0.84	1.87	2.700 (3)	168.
O3W—H3W1···O1^iii^	0.84	1.92	2.723 (3)	160.
O3W—H3W2···O4	0.84	1.85	2.688 (3)	175.
O4W—H4W1···O3	0.85	1.89	2.734 (3)	174.
O4W—H4W2···O7^iv^	0.85	1.88	2.704 (3)	162.
C13—H13···O9	0.95	2.31	3.031 (5)	133.

Symmetry codes: (i) −*x*+1, −*y*+1, −*z*; (ii) *x*−1, *y*, *z*; (iii) −*x*+1, −*y*, −*z*+1; (iv) −*x*+2, −*y*+1, −*z*.

## References

[bb1] Brandenburg, K. (2008). *DIAMOND* Crystal Impact GbR, Bonn, Germany.

[bb2] Burla, M. C., Caliandro, R., Camalli, M., Carrozzini, B., Cascarano, G. L., De Caro, L., Giacovazzo, C., Polidori, G. & Spagna, R. (2005). *J. Appl. Cryst.* **38**, 381–388.

[bb3] Dobosz, A., Dudarenko, N. M., Fritsky, I. O., Głowiak, T., Karaczyn, A., Kozłowski, H., Sliva, T. Yu. & Świątek-Kozłowska, J. (1999). *J. Chem. Soc. Dalton Trans* pp. 743–749.

[bb4] Fritsky, I. O., Kozłowski, H., Sadler, P. J., Yefetova, O. P., Świątek-Kozłowska, J., Kalibabchuk, V. A. & Głowiak, T. (1998). *J. Chem. Soc. Dalton Trans.* pp. 3269–3274.

[bb5] Fritsky, I. O., Ott, R., Pritzkow, H. & Krämer, R. (2001). *Chem. Eur. J.* **7**, 1221–1231. 10.1002/1521-3765(20010316)7:6<1221::aid-chem1221>3.0.co;2-t 11322548

[bb6] Hynes, J. B. (1970). *J. Med. Chem.* **13**, 1235–1237. 10.1021/jm00300a056 5479878

[bb7] Kovbasyuk, L., Pritzkow, H., Krämer, R. & Fritsky, I. O. (2004). *Chem. Commun* pp. 880–881. 10.1039/b316225g 15045110

[bb8] Krämer, R. & Fritsky, I. O. (2000). *Eur. J. Org. Chem.* pp. 3505–3510.

[bb9] Mokhir, A. A., Gumienna-Kontecka, E. S., Świątek-Kozłowska, J., Petkova, E. G., Fritsky, I. O., Jerzykiewicz, L., Kapshuk, A. A. & Sliva, T. Yu. (2002). *Inorg. Chim. Acta*, **329**, 113–121.

[bb10] Moroz, Y. S., Szyrweil, L., Demeshko, S., Kozłowski, H., Meyer, F. & Fritsky, I. O. (2010). *Inorg. Chem* **49**, 4750-4752. 10.1021/ic100555s 20441208

[bb11] Nonius (2000). *COLLECT* Nonius BV, Delft, The Netherlands.

[bb12] Otwinowski, Z. & Minor, W. (1997). *Methods in Enzymology*, Vol. 276, *Macromolecular Crystallography*, Part A, edited by C. W. Carter Jr & R. M. Sweet, pp. 307–326. New York: Academic Press.

[bb13] Sachse, A., Penkova, L., Noel, G., Dechert, S., Varzatskii, O. A., Fritsky, I. O. & Meyer, F. (2008). *Synthesis*, **5**, 800–806.

[bb14] Sheldrick, G. M. (2008). *Acta Cryst.* A**64**, 112–122. 10.1107/S0108767307043930 18156677

[bb15] Sliva, T. Yu., Kowalik-Jankowska, T., Amirkhanov, V. M., Głowiak, T., Onindo, C. O., Fritsky, I. O. & Kozłowski, H. (1997). *J. Inorg. Biochem.* **65**, 287–294.

[bb16] Świątek-Kozłowska, J., Fritsky, I. O., Dobosz, A., Karaczyn, A., Dudarenko, N. M., Sliva, T. Yu., Gumienna-Kontecka, E. & Jerzykiewicz, L. (2000). *J. Chem. Soc. Dalton Trans* pp. 4064–4068.

[bb17] Wörl, S., Fritsky, I. O., Hellwinkel, D., Pritzkow, H. & Krämer, R. (2005*b*). *Eur. J. Inorg. Chem* pp. 759–765.

[bb18] Wörl, S., Pritzkow, H., Fritsky, I. O. & Krämer, R. (2005*a*). *Dalton Trans* pp. 27–29. 10.1039/b417053a 15605143

